# Prognostic value of microRNAs in hepatocellular carcinoma: a meta-analysis

**DOI:** 10.18632/oncotarget.20883

**Published:** 2017-09-14

**Authors:** Yue Zhang, Chao Wei, Cong-Cong Guo, Rong-Xiu Bi, Jin Xie, Dong-Hui Guan, Chuan-Hua Yang, Yue-Hua Jiang

**Affiliations:** ^1^ First Clinical Medical College, Shandong University of Traditional Chinese Medicine, Jinan 250355, Shandong, People’s Republic of China; ^2^ Department of Orthopedics, Affiliated Hospital of Shandong University of Traditional Chinese Medicine, Jinan 250011, Shandong, People’s Republic of China; ^3^ Department of Cardiology, Affiliated Hospital of Shandong University of Traditional Chinese Medicine, Jinan 250011, Shandong, People’s Republic of China; ^4^ Central Laboratory, Affiliated Hospital of Shandong University of Traditional Chinese Medicine, Jinan 250011, Shandong, People’s Republic of China

**Keywords:** microRNA, hepatocellular carcinoma, prognosis, meta-analysis

## Abstract

**Background:**

Numerous articles reported that dysregulated expression levels of miRNAs correlated with survival time of HCC patients. However, there has not been a comprehensive meta-analysis to evaluate the accurate prognostic value of miRNAs in HCC.

**Design:**

Meta-analysis.

**Materials and Methods:**

Studies, published in English, estimating expression levels of miRNAs with any survival curves in HCC were identified up until 15 April, 2017 by performing online searches in PubMed, EMBASE, Web of Science and Cochrane Database of Systematic Reviews by two independent authors. The pooled hazard ratios (HR) with 95% confidence intervals (CI) were used to estimate the correlation between miRNA expression and overall survival (OS).

**Results:**

54 relevant articles about 16 miRNAs, with 6464 patients, were ultimately included. HCC patients with high expression of tissue miR-9 (HR = 2.35, 95% CI = 1.46–3.76), miR-21 (HR = 1.76, 95% CI = 1.29–2.41), miR-34c (HR = 1.64, 95% CI = 1.05–2.57), miR-155 (HR = 2.84, 95% CI = 1.46–5.51), miR-221 (HR = 1.76, 95% CI = 1.02–3.04) or low expression of tissue miR-22 (HR = 2.29, 95% CI = 1.63–3.21), miR-29c (HR = 1.35, 95% CI = 1.10–1.65), miR-34a (HR = 1.84, 95% CI = 1.30–2.59), miR-199a (HR = 2.78, 95% CI = 1.89–4.08), miR-200a (HR = 2.64, 95% CI = 1.86–3.77), miR-203 (HR = 2.20, 95% CI = 1.61–3.00) have significantly poor OS (*P* < 0.05). Likewise, HCC patients with high expression of blood miR-21 (HR = 1.73, 95% CI = 1.07–2.80), miR-192 (HR = 2.42, 95% CI = 1.15–5.10), miR-224 (HR = 1.56, 95% CI = 1.14–2.12) or low expression of blood miR-148a (HR = 2.26, 95% CI = 1.11–4.59) have significantly short OS (*P* < 0.05).

**Conclusions:**

In conclusion, tissue miR-9, miR-21, miR-22, miR-29c, miR-34a, miR-34c, miR-155, miR-199a, miR-200a, miR-203, miR-221 and blood miR-21, miR-148a, miR-192, miR-224 demonstrate significantly prognostic value. Among them, tissue miR-9, miR-22, miR-155, miR-199a, miR-200a, miR-203 and blood miR-148a, miR-192 are potential prognostic candidates for predicting OS in HCC.

## INTRODUCTION

Numerous studies reported expression levels of tissue [1–194] or blood [195–221] miRNAs were related with prognosis of HCC patients. HCC is one of the most common tumors, over 700,000 new cases are reported yearly, and HCC is considered as the third primary etiology of tumor-associated mortality rate globally [222–224]. In spite of enormous process in diagnosis and comprehensive therapy over the last few decades, HCC patients still have poor prognosis, primarily due to its high rate of recurrence [225] and metastasis [226].

miRNAs, a cluster of endogenous short non-coding single strand RNAs, serve as significant post-transcriptional regulatory factor of genetic expression via interacting with the 3′-UTR of the targeted mRNAs [227]. Conspicuously, due to widespread RNAase in the blood environment, circulating miRNAs displayed predominant stability. As a noninvasive detection method, circulating miRNA (blood) demonstrated more potential value as diagnostic and prognostic biomarkers than tissue miRNAs. Studies [228, 229] conducted in preclinical models and cancer patients proved that malignant tumor influences expression levels of miRNAs in the blood and that certain serum miRNAs are correlated with particular cancers. Though the way requires more validation, the finding possibly discloses the avenue to a creative method of detecting cancers via measurement of serum or plasma miRNAs.

Thus far, substantial investigations have discovered that miRNAs are involved and play a crucial role in the carcinogenesis of HCC [230, 231] while some miRNAs are up-regulated and others down-regulated in HCC. For example, Wong et al. [232] gained contrasting results that identifiable difference in miRNA expression pattern could not be discovered between primary HCC and venous metastases. However, comparing venous metastases to primary HCC, a prominent universal decrease of miRNA expression levels was assayed. Their results indicated that miRNA abnormality relatively early occured in liver carcinogenesis and the later universally decreased miRNA aggravated the preexisting miRNA abnormity to further accelerate HCC metastasis.

Nevertheless, there has not been a synthetic meta-analysis to assess precise prognostic value of miRNAs in HCC. As a consequence, it is of vital significance to develop a meta-analysis with an aim to evaluate it.

## RESULTS

### Study selection

**Figure 1:**
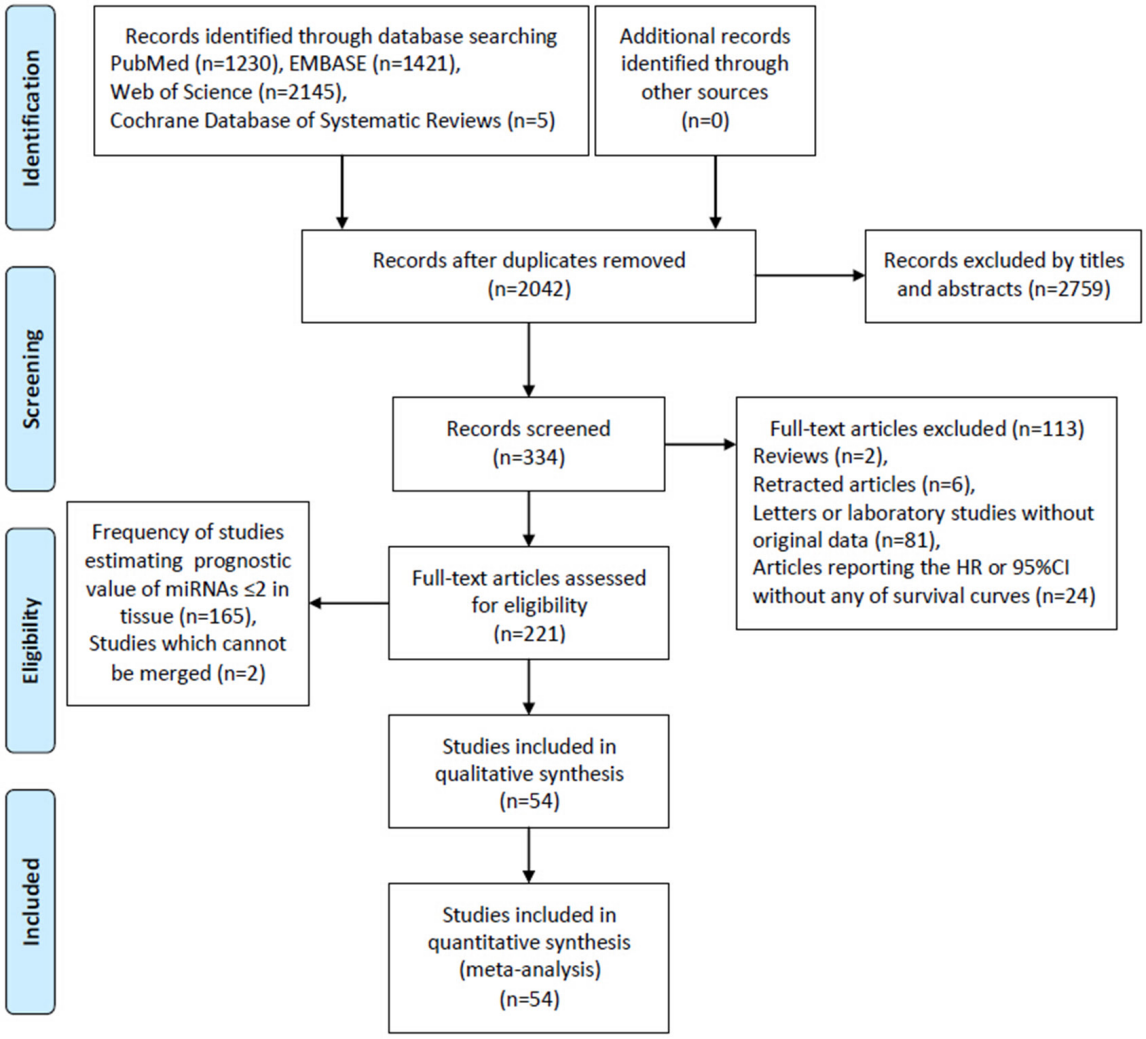
Flow diagram of literature search and selection

Figure 1 showed a flow chart with details about the study selection process.

### Study frequency

Tables 1 (tissue) and 2 (blood) showed the frequency of researches evaluating prognostic value of miRNAs, including miRNA name, number of investigations assessing prognostic value, and reference.

**Table 1:** Frequency of studies estimating prognostic value of tissue miRNA expression in hepatocellular carcinoma

miR	N	R	miR	N	R	miR	N	R	miR	N	R	miR	N	R	miR	N	R
1	1	1	29b	2	38, 39	125a	1	70	192	2	107, 108	330	1	143	520g	1	167
7	1	2	29c	3	38, 40, 41	125b	2	71, 72	193a	1	29	331-3p	1	144	522	1	168
9–1	2	3, 4	30a-5p	1	42	126	1	73	193b	1	29	338-3p	1	145	542-5p	1	169
9–2	2	3, 4	30a	1	43	128-3p	1	74	194	1	109	339-5p	1	146	545	1	170
9	3	5–7	30b-5p	1	44	129-5p	1	75	195	2	110, 111	365	1	147	589-5p	1	171
10b	1	8	30b	1	9	129–2	1	76	197	1	112	370	1	148	592	1	172
15a	1	9	30c	1	9	130a	1	77	199a-5p	2	11, 113	372	2	149, 150	608	1	173
15b	1	9	30d	1	3	130b	1	78	199a^*^	1	114	375	1	151	610	1	174
17-5p	1	10	31	1	45	135a	2	18, 79	199a	1	114	381	1	9	622	1	175
18a	1	11	33a-3p	1	46	137	2	80, 81	199b-5p	1	115	383	1	152	625	1	176
18b	1	12	34a-5p	1	47	139-5p	1	82	200a	3	116–118	421	1	141	630	1	177
19a	1	13	34a	3	48–50	139	1	83	203	3	119–121	424	2	141, 153	634	1	178
19b	1	14	34b	2	50, 51	140-5p	1	84	204	1	107	425-3p	1	154	638	2	179, 180
20a	2	15, 16	34c-3p	1	52	145	1	85	205	1	122	429	1	155	744	1	181
20b	1	17	34c	2	50, 51	146a	1	86	210	2	123, 124	432	1	9	876-5p	1	9
21a	1	18	92a	1	53	148a	3	87–89	211	1	125	451	1	156	885-5p	1	182
21	8	2, 19–25	93	1	54	148b	2	90, 91	212	2	126, 127	452	1	157	892a	1	183
22	3	4, 26, 27	98	1	55	149	2	92, 93	214	2	114, 128	454	1	158	940	2	184, 185
23a	2	28, 29	99a	2	56, 57	150	1	95	216b	1	129	455	1	159	944	1	186
23b	1	29	99b	1	58	151	1	96	218	1	130	486-3p	1	9	1180	1	187
24	1	30	100	2	59, 60	152	1	97	219-5p	1	131	486-5p	1	160	1246	1	188
25	2	31, 32	101	2	61, 62	155-3p	1	98	221	5	20, 132–135	489	1	161	1268a	1	189
26a	1	33	103	1	2	155	3	9, 99, 100	222	1	136	494	1	162	1269	1	190
26b-5p	1	34	105–1	1	63	182	1	101	224	1	137	497	1	163	1323	1	191
27b	1	35	106b	2	64, 65	183	1	102	296	1	138	503	1	164	3127	1	192
28-3p	1	36	107	1	2	185	1	103	302d	1	139	511–1	1	3	3677	2	3, 141
28-5p	1	36	122	2	66, 67	187-3p	1	104	325	1	140	511–2	1	3	4458	1	193
29a-5p	1	37	124–1	1	68	188-5p	1	105	326	2	3, 141	511	1	141	4782-3p	1	194
29a	2	9, 38	124	1	69	191	1	106	329	1	142	519a	2	165, 166			

Highlighted studies were included in the present meta-analysis; N: Number of studies estimating prognostic value; R: References.

**Table 2:** Frequency of studies estimating prognostic value of blood miRNA expression in hepatocellular carcinoma

miR	N	R	miR	N	R	miR	N	R
1	1	195	139-5p	1	209	218	1	216
10b-3p	1	196	148a	2	208, 211	221	1	217
21	4	197–200	148b	1	211	224-5p	1	198
24-3p	1	201	150	1	212	224	1	218
26a	1	200	152	1	211	311-3p	1	214
29a-3p	1	202	181a-5p	1	213	335	1	219
29a	1	200	182	1	214	422a	1	209
96	1	203	192-5p	1	202	424	1	220
101	1	204	192	1	208	486-5p	1	209
122	6	195, 198, 205–208	200a	1	198	1246	1	208
125b	1	209	210	1	215	1290	1	208
128-2	1	210	215	1	208	4463	1	221

Highlighted studies were included in the present meta-analysis; N: Number of studies estimating prognostic value; R: References.

### Study characteristics

Supplementary Table 1 comprehensively presented the characteristics and details (names of miRNAs, information about the included articles, detected samples, sample size, stage, cut-off value, detection methods, follow-up, survival outcome with HR and 95% CI) of studies with Kaplan-Meier survival curves (K-M curves) in HCC. If the survival outcome was not furnished directly and merely as K-M curves, we used the software Engauge Digitizer version 4.1 [233] to extract the data from K-M curves. Additionally, if both the univariate and multivariate outcomes were covered, we just chose the latter in that the confounding factors were corrected.

### Meta-analysis

Table 3 presented a summary of the HR estimated from pooled analysis for the included miRNAs. A total of 16 miRNAs were screened by our present study.

**Table 3:** Summary of the HR for miRNA expression in hepatocellular carcinoma

miRNA	Survival analysis	Number of articles	Included studies	HR	95% CI	Figure	*P* value	Heterogeneity (Higgins I^2^ statistic)	Total patients
High miR-9	OS	2	5, 7	2.35	1.46–3.76	4	< 0.01	I^2^ = 35.3%, *P =* 0.21	320
High miR-9	DFS/RFS	2	6,7	2.49	1.57–3.97	4	< 0.01	I^2^ = 0.0%, *P =* 0.80	180
High miR-21	OS	6	19, 20, 22–25	1.76	1.29–2.41	2A	< 0.01	I^2^ = 17.1%, *P =* 0.30	461
High miR-21	DFS	3	2, 21, 22	3.48	1.89–6.44	2A	< 0.01	I^2^ = 40.4%, *P =* 0.19	274
High miR-21	OS^m^	2	22, 23	2.72	1.49–4.95	2A	< 0.01	I^2^ = 0.0%, *P =* 0.58	231
High miR-21	RFS/DFS	2	197, 200	1.11	0.62–1.96	7	0.73	I^2^ = 72.7%, *P =* 0.06	246
High miR-21	OS	2	198, 199	1.73	1.07–2.80	7	0.03	I^2^ = 59.0%, *P =* 0.12	233
Low miR-22	OS	2	4, 27	2.29	1.63–3.21	4	< 0.01	I^2^ = 0.0%, *P =* 0.85	564
Low miR-29a	OS	3	9, 37, 38	1.29	0.91–1.81	4	0.15	I^2^ = 47.8%, *P =* 0.15	657
Low miR-29a	RFS	2	9, 37	0.82	0.38–1.77	4	0.61	I^2^ = 83.0%, *P =* 0.02	434
High miR-29a	PFS/DFS	2	200, 202	1.12	0.32–3.94	7	0.86	I^2^ = 89.1%, *P* < 0.01	194
Low miR-29c	OS	3	38, 40, 41	1.35	1.10–1.65	4	< 0.01	I^2^ = 0.0%, *P =* 0.47	467
Low miR-34a	OS	4	47–50	1.84	1.30–2.59	5	< 0.01	I^2^ = 48.7%, *P =* 0.12	339
Low miR-34a	PFS/RFS/DFS	3	47, 49, 50	1.43	1.17–1.74	5	< 0.01	I^2^ = 20.3%, *P =* 0.29	309
High miR-34c	OS	2	50, 52	1.64	1.05–2.57	5	0.03	I^2^ = 0.0%, *P =* 0.41	156
High miR-34c	DFS	3	50–52	1.15	0.72–1.85	5	0.56	I^2^ = 72.5%, *P =* 0.03	236
High miR-122	OS	6	195, 198, 205–208	0.89	0.49–1.60	3A	0.69	I^2^ = 84.8%, *P* < 0.01	896
High miR-122	DFS	2	205, 208	1.62	0.40–6.41	3A	0.50	I^2^ = 50.6%, *P =* 0.16	182
Low miR-148a	OS	2	87, 88	1.83	0.80–4.20	5	0.15	I^2^ = 67.2%, *P* < 0.05	356
Low miR-148a	RFS	3	87–89	1.37	0.99–1.91	5	0.06	I^2^ = 0.0%, *P =* 0.55	445
Low miR-148a	OS	2	208, 211	2.26	1.11–4.59	7	0.03	I^2^ = 0.0%, *P =* 0.99	138
High miR-155	OS	3	98–100	2.84	1.46–5.51	5	< 0.01	I^2^ = 57.8%, *P =* 0.09	269
High miR-155	RFS/DFS	3	9, 99, 100	2.09	1.56–2.79	5	< 0.01	I^2^ = 0.0%, *P =* 0.66	440
High miR-192	OS	2	202, 208	2.42	1.15–5.10	7	0.02	I^2^ = 6.7%, *P =* 0.30	136
High miR-192	PFS/DFS	2	202, 208	1.97	0.96–4.03	7	0.06	I^2^ = 2.5%, *P =* 0.31	136
Low miR-199a	OS	2	113, 114	2.78	1.89–4.08	6	< 0.01	I^2^ = 0.0%, *P =* 0.38	239
Low miR-200a	OS	3	116–118	2.64	1.86–3.77	6	< 0.01	I^2^ = 0.0%, *P =* 0.91	336
Low miR-203	OS	2	119, 121	2.20	1.61–3.00	6	< 0.01	I^2^ = 0.0%, *P =* 0.45	204
Low miR-203	RFS	2	119, 120	2.12	0.40–11.16	6	0.37	I^2^ = 76.1%, *P =* 0.04	161
High miR-221	OS	3	20, 132, 135	1.76	1.02–3.04	6	0.04	I^2^ = 67.6%, *P* < 0.05	240
High miR-221	RFS/MFS/DFS	4	132–135	2.26	1.53–3.35	6	< 0.01	I^2^ = 50.4%, *P =* 0.09	334
High miR-224	OS	2	198, 218	1.56	1.14–2.12	7	< 0.01	I^2^ = 24.2%, *P =* 0.25	318

HR: hazard ratios; CI: confidence intervals; OS: overall survival; DFS: disease-free survival; RFS: recurrence-free survival; PFS: progression-free survival; MFS: metastasis-free survival; ^m^Multivariate analysis.

### Significantly prognostic value of high tissue miR-21 expression in OS

**Figure 2:**
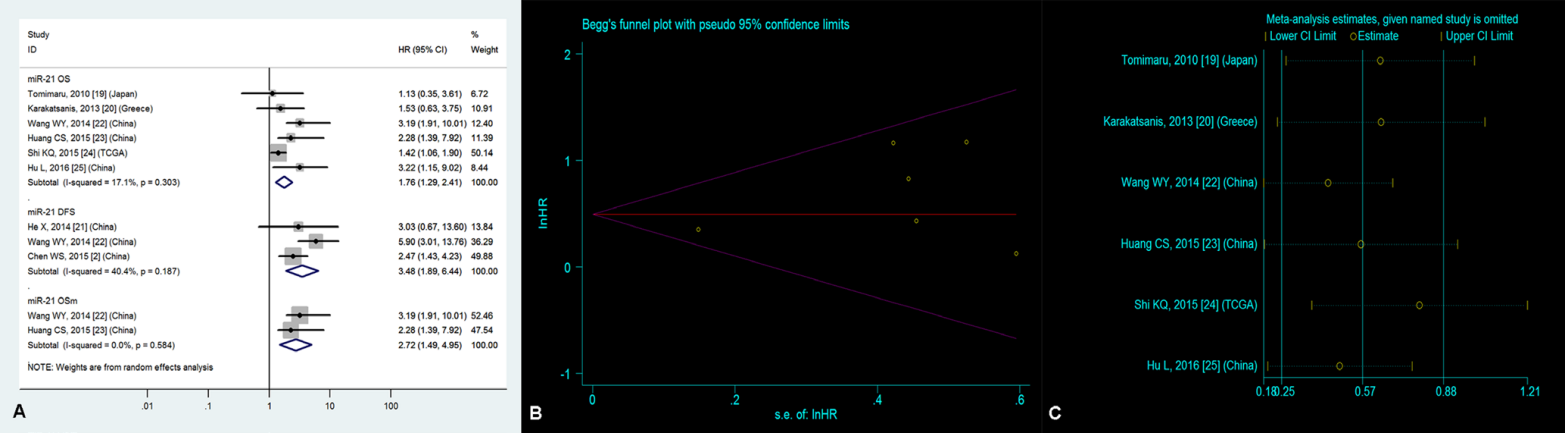
(**A**) Forest plot of the analyses about high expression of tissue miR-21 and OS, DFS or OS (multivariate analysis); (**B**) Publication bias of the analysis about high expression of tissue miR-21 and OS and (**C**) Sensitivity analysis of the study about high expression of tissue miR-21 and OS.

Six studies [19, 20, 22–25] focused on the correlation between high tissue miR-21 level and OS, suggesting that HCC patients with high tissue miR-21 level demonstrated a significantly worse OS than those with low tissue miR-21 level (HR = 1.76, 95% CI = 1.29–2.41, *P* < 0.01, Figure 2A).

### Publication bias

For the purpose of evaluating publication bias on OS of HCC patients with high tissue miR-21 level, we employed the Begg’s funnel plot (Figure 2B). Accordingly, the *P* value was 0.21, suggesting nonexistent publication bias.

### Sensitivity analysis

The sensitivity analysis did not manifest variances among the outcomes in terms of the exclusion of any single research (Figure 2C) in the estimation on OS of HCC patients with high tissue miR-21 level, indicating that no individual investigation significantly affected the merged HR with 95% CI.

### No significantly prognostic value of high blood miR-122 expression in OS

**Figure 3:**
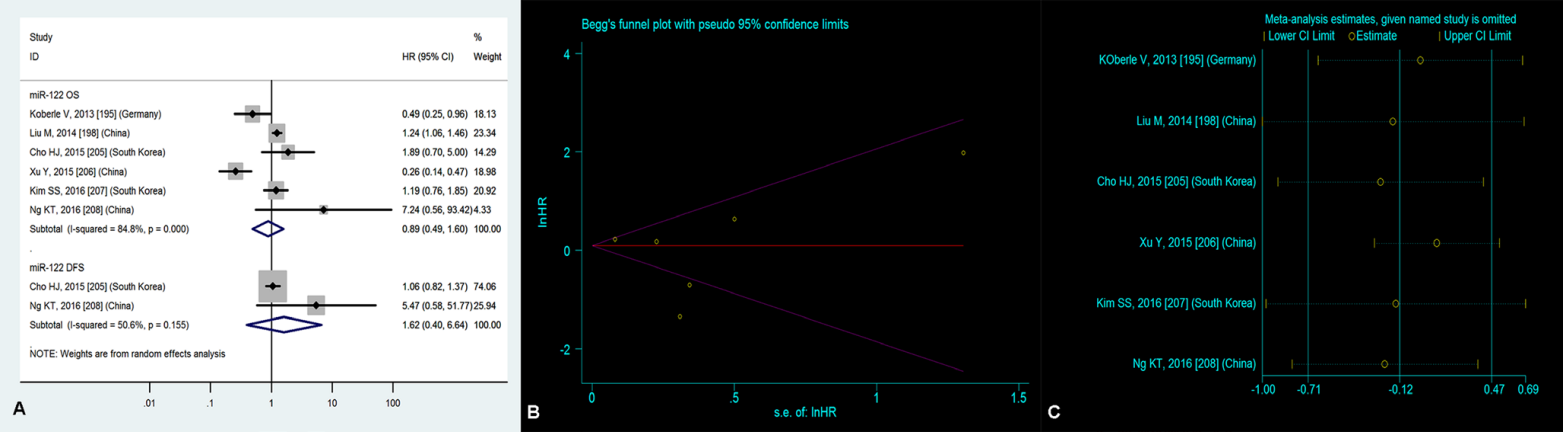
(**A**) Forest plot of the analyses about high expression of blood miR-122 and OS or DFS; (**B**) Publication bias of the analysis about high expression of blood miR-122 and OS and (**C**) Sensitivity analysis of the study about high expression of blood miR-122 and OS.

Six researches [1, 4, 11–14] concentrated on the relationship between high blood miR-122 level and OS, manifesting that there was no significant correlation between high blood miR-122 level and OS (HR = 0.89, 95% CI = 0.49–1.60, *P* = 0.69, Figure 3A).

### Publication bias

For the sake of estimating publication bias on OS of HCC patients with high blood miR-122 level, we employed the Begg’s funnel plot (Figure 3B). Consequently, the *P* value was 0.56, suggesting nonexistent publication bias.

### Sensitivity analysis

The sensitivity analysis did not manifest variances among the outcomes in terms of the exclusion of any single research (Figure 3C) in the estimation on OS of HCC patients with high blood miR-122 level, indicating that no individual investigation significantly affected the merged HR with 95% CI.

### Meta-regression

We employed the meta-regression to seek source of heterogeneity (I^2^ = 84.8%) on OS of HCC patients with high blood miR-122 level. The details were shown in Table 4, and source of heterogeneity was significantly caused by maximum months of follow-up (*P* = 0.01).

**Table 4:** Results of meta-regression on OS of blood miR-122 expression in hepatocellular carcinoma

Variables	Details	tau^2^	I^2^ (%)	Adj R^2^ (%)	*P* value
Year	2013–2016	0.73	87.82	–29.29	0.46
Country	Germany, China, South Korea	0.50	86.34	11.27	0.34
Design	Prospective, Retrospective	0.61	88.00	–21.49	0.51
Sample	Serum, Plasma	0.45	87.43	20.85	0.16
Number	295, 136, 120, 122, 161, 62	0.69	86.03	–21.90	0.47
Stage	None, I–IV	0.52	87.59	8.14	0.39
Method	qRT-PCR, RT-qPCR	0.51	87.01	10.36	0.22
Follow-up	26, 48, 96, 40, 79, 125	0.28	84.28	50.82	0.08
Follow-up	< 48, ≥ 48	0.00	9.67	100.00	0.01

### Tissue miR-9, miR-22, miR-29c, miR-34a, miR-34c, miR-155, miR-199a, miR-200a, miR-203, miR-221 and blood miR-21, miR-148a, miR-192, miR-224 have significantly prognostic values in OS

**Figure 4:**
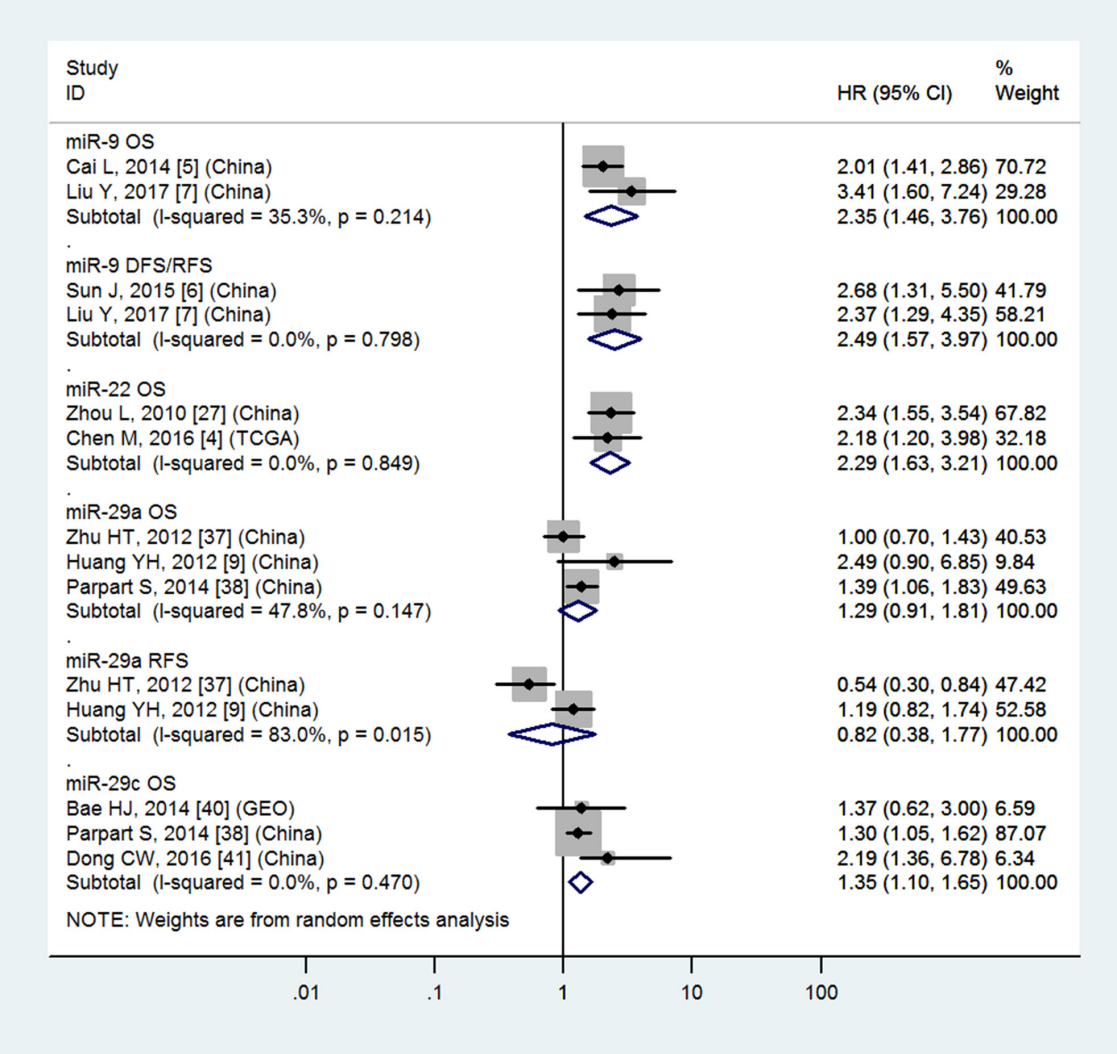
Forest plot of the analyses about high expression of tissue miR-9 or low expression of tissue miR-22, 29a, 29c and OS, DFS/RFS or RFS

**Figure 5:**
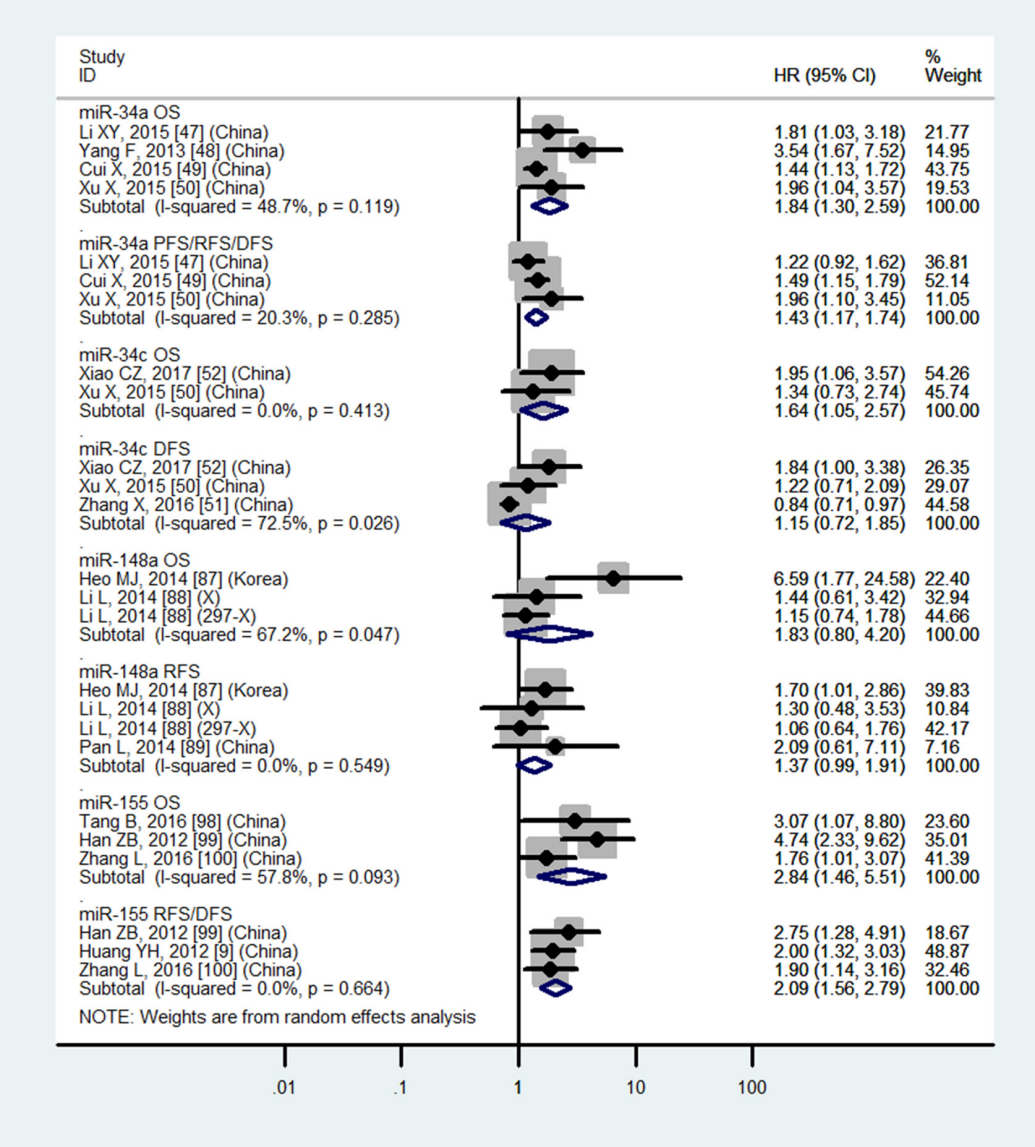
Forest plot of the analyses about high expression of tissue miR-34c, 155 or low expression of tissue miR-34a, 148a and OS, PFS/RFS/DFS, DFS, RFS or RFS/DFS

**Figure 6:**
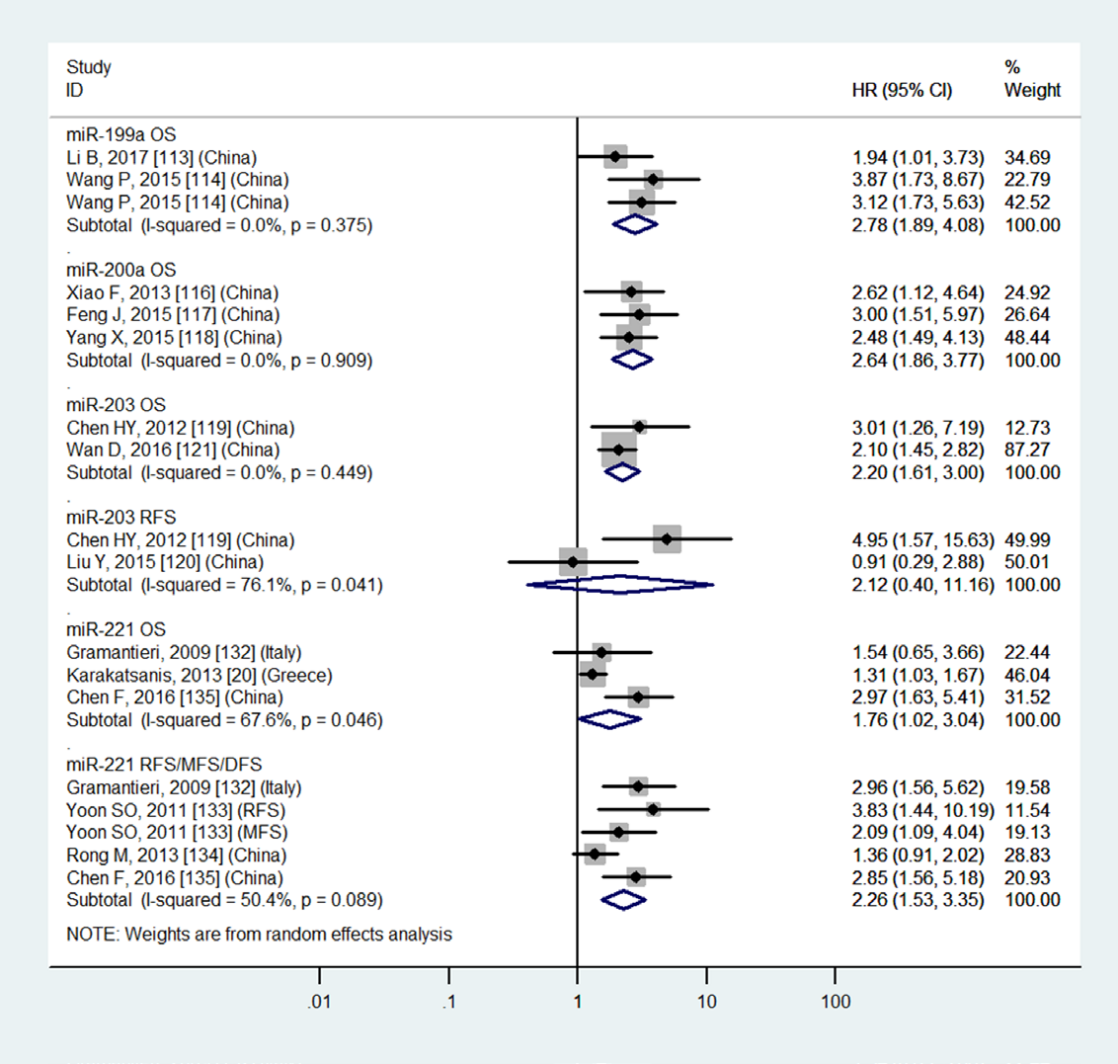
Forest plot of the analyses about high expression of tissue miR-221 or low expression of tissue miR-199a, 200a, 203 and OS, RFS or RFS/MFS/DFS

**Figure 7:**
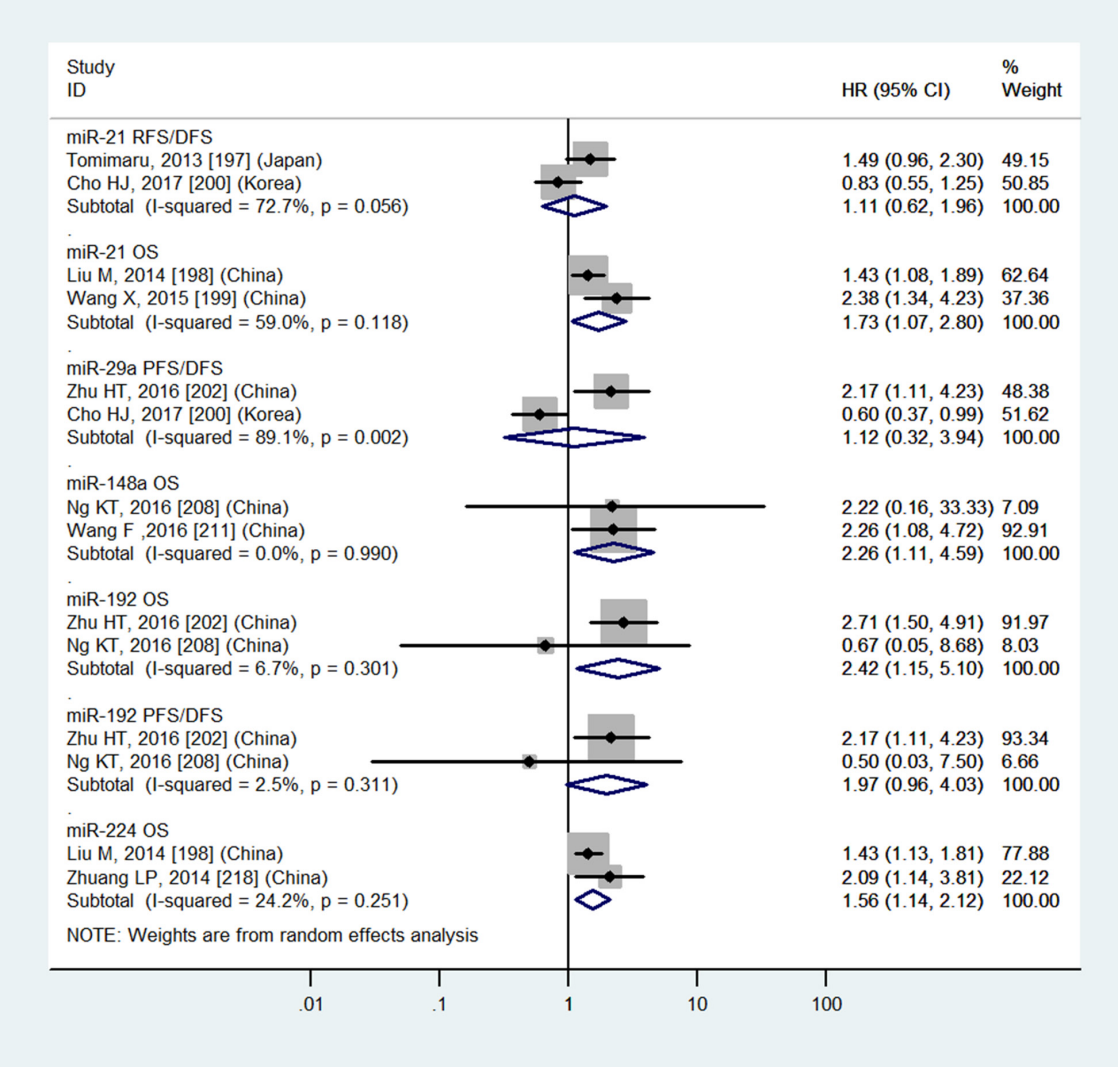
Forest plot of the analyses about high expression of blood miR-21, 29a, 192, 224 or low expression of blood miR-148a and RFS/DFS, OS or PFS/DFS

Table 3 and Figures 4–7 showed the details.

### Tissue miR-29a, miR-148a and blood miR-29a do not have significantly prognostic values in OS

Table 3 and Figures 4, 5 and 7 showed the details.

## DISCUSSION

### Status quo

Numerous articles reported that dysregulated expression levels of miRNAs correlated with survival time of HCC patients [1–221]. Nevertheless, there has not been a comprehensive meta-analysis to assess the accurate prognostic value of miRNAs in HCC. Therefore, it was conducted to estimate the relationship between dysregulated miRNA level and survival time of HCC patients.

### Main discoveries

For HCC patients, tissue miR-9, miR-21, miR-22, miR-29c, miR-34a, miR-34c, miR-155, miR-199a, miR-200a, miR-203, miR-221 and blood miR-21, miR-148a, miR-192, miR-224 demonstrate significantly prognostic value (*P* < 0.05). In the light of our reference standard, tissue miR-9, miR-22, miR-155, miR-199a, miR-200a, miR-203 and blood miR-148a, miR-192 potential prognostic candidates for predicting the OS of HCC patients (HR ≥ 2).

### Molecular mechanisms for included miRNAs

For included miRNAs in the current study, a summary of miRNAs with changed levels, their possible targets and pathways enrolled in the present study has been presented in Table 5. From the data of the table, these potential targets and pathways may be involved with survival outcome of HCC patients.

**Table 5:** Summary of miRNAs with altered expression, their potential targets and pathways entered this study

miRNA	Reference	Expression	Potential target	Pathway
9	5–7	Up	GALNT4	None
21	2, 19–25, 197–200	Up	None	None
22	4, 26, 27	Down	YWHAZ, HDAC4	Cell invasion, migration, proliferation, tumourigenicity and YWHAZ/AKT1/foxo3a signaling
29a	9, 37, 38, 200, 202	Up	None	None
29c	38, 40, 41	Down	SIRT1	None
34a	47–50	Down	FOXM1, MYC, BCL2, AXL	Cell apoptosis, chemoresistance, proliferation, viability and FOXM1/MYC signaling
34c	50–52	Down	NCKAP1	Cell cycle, growth, invasion and proliferation
122	195, 198, 205–208	None	None	None
148a	87-89, 208, 211	Down	USP4, SIP1	Cell invasion, migration and proliferation
155	9, 98–100	Up	ARID2, FBXW7	Cell apoptosis, cycle, invasion, proliferation and tumorigenesis
192	202, 208	Up	None	None
199a	11, 113, 114	Down	HIF1A, VEGFA, IGF1, IGF2	Cell growth, invasion, proliferation and Warburg effect
200a	116-118	Down	MACC1, CDK6, ZEB2	Cell cycle, growth, metastasis and proliferation
203	119–121	Down	ADAM9, HULC	Cell apoptosis, invasion and proliferation
221	20, 132–135	Up	Bmf	Cell apoptosis and growth
224	198, 218	None	None	None

GALNT4: polypeptide N-acetylgalacosaminyltransferase 4; YWHAZ: tyrosine 3-monooxygenase/tryptophan 5-monooxygenase activation protein zeta; HDAC4: histone deacetylase 4; AKT1: AKT serine/threonine kinase 1; foxo3a: forkhead box O3A; SIRT1: sirtuin 1; FOXM1: forkhead box M1; MYC: MYC proto-oncogene, bHLH transcription factor; BCL2: BCL2, apoptosis regulator; AXL: AXL receptor tyrosine kinase; NCKAP1: NCK associated protein 1; USP4: ubiquitin specific peptidase 4; SIP1: Sip1p; ARID2: AT-rich interaction domain 2; FBXW7: F-box and WD repeat domain containing 7; HIF1A: hypoxia inducible factor 1 alpha subunit; VEGFA: vascular endothelial growth factor A; IGF1: insulin like growth factor 1; IGF2: insulin like growth factor 2; MACC1: MACC1, MET transcriptional regulator; CDK6: cyclin dependent kinase 6; ZEB2: zinc finger E-box binding homeobox 2; ADAM9: ADAM metallopeptidase domain 9; HULC: hepatocellular carcinoma up-regulated long non-coding RNA; BMF: Bcl2 modifying factor.

### Strengths of the meta-analysis

There are a few strengths in this study, which are as follows: (1) nearly all articles estimating associations between miRNA level and survival result of HCC patients are shown in the current meta-analysis; (2) the number about HCC patients of all researches included in this study are more than or equal to 30, which makes the meta-analyses more convincing; (3) the Begg’s funnel plot and sensitivity analysis were used for miR-21 and miR-122, which excluded publication bias and excessive influence of individual study; (4) we employed meta-regression to seek source of heterogeneity, which indicated that months of follow-up were significantly associated with it; (5) studies merely proposing HR or 95% CI without K-M curves were excluded.

### Limitations

Simultaneously, there are also limitations for the current work: (1) only English articles were included by us, which possibly excluded some studies written in other languages; (2) not all the articles assessing associations between miRNA level and survival time were included in the present study, which might neglect some potential miRNAs; (3) a few variables emerged among the included investigations, including different kinds of samples from HCC patients at different stages, cut-off values and detection methods, and only random-effects models were employed for all meta-analyses; (4) although overall studies included 54 relevant articles and 6464 patients in the present study, the number of articles and patients may be not enough for 16 miRNAs focused on.

### Implications for future clinical and scientific research

With expression profiles shown in Tables 1 and 2, we can conveniently find relevant article about a single miRNA. Thus, the present study tendency for miRNAs in HCC can be easily obtained by basic researchers. Meanwhile, combined detection of multi-miRNAs can greatly increase the predict level for HCC patients. Besides, for clinical doctors, combined use of tissue and blood from HCC patients can bring about synergistic effect to estimation of prognosis.

## MATERIALS AND METHODS

### Search strategy, inclusion criteria and exclusion criteria

The details were presented in Table 6. Two authors (Yue Zhang and Chao Wei) independently performed this comprehensive online search.

**Table 6:** Information of search methods and criteria of inclusion and exclusion

Methods	Information
Search strategy	4 search engines, including PubMed, EMBASE, Web of Science and Cochrane Database of Systematic Reviews
Search deadline	15 April, 2017
Search terms	mir and hepatocellular carcinoma
Inclusion criteria	(1) Patients with hepatocellular carcinoma; (2) Expression of miRNAs and survival outcome in tissue, plasma or serum were measured; (3) At least, one of survival curves about overall survival (OS), cause-specific survival (CSS), disease-free survival (DFS), recurrence-free survival (RFS), progression-free survival (PFS) and metastasis-free survival (MFS) was measured, with or without the HR or 95% CI; (4) Full text articles published in English
Exclusion criteria	(1) Reviews, letters or laboratory studies without original data and retracted articles; (2) Frequency of studies estimating prognostic value of miRNAs ≤ 2 in tissue; (3) Studies which cannot be merged; (4) If more than one article had been published on the identical study cohort, only the most comprehensive study was selected for the present meta-analysis

### Quality assessment

Yue Zhang and Chao Wei confirmed all eligible investigations that analyzed the prognostic value of miRNAs in HCC, and Yue-Hua Jiang reassessed uncertain data.

### Statistical analysis

All analyses were conducted using Stata version 13.0 (StataCorp, College Station, Texas, USA). The relative effect sizes for HR were characterized as moderate (protective [0.51–0.75] or contributory [1.35–1.99]) and large (≤ 0.50 or ≥ 2). The HR was considered significant at the *P* < 0.05 level if the 95% CI did not include the value 1. If the *P* values from OS and other survival results about corresponding miRNAs were inconsistent, the HR from OS was considered to the main reference standard. Because different types of samples (tissue, plasma and serum) from HCC patients at different disease stages, cut-off values and miRNA methods were used in individual studies, random-effects models (DerSimonian-Laird method) were more appropriate than fixed-models (Mantel-Haenszel method) for most of the analyses. Consequently, the random-effects models were used in the current meta-analysis. Source of heterogeneity was explored through meta-regression. Publication bias was estimated using the Begg’s funnel plot. A two-tailed *P* value < 0.05 was considered significant. Sensitivity analysis (influence analysis) was carried out to test how powerful the combined effect size was to removal of individual investigation. If the point assessment was out of the 95% CI of the pooled effect size after it was removed from the analysis, an individual study was doubted to have excessive influence.

## CONCLUSIONS

In conclusion, tissue miR-9, miR-21, miR-22, miR-29c, miR-34a, miR-34c, miR-155, miR-199a, miR-200a, miR-203, miR-221 and blood miR-21, miR-148a, miR-192, miR-224 demonstrate significantly prognostic value. Among them, tissue miR-9, miR-22, miR-155, miR-199a, miR-200a, miR-203 and blood miR-148a, miR-192 are potential prognostic candidates for predicting OS in HCC.

## SUPPLEMENTARY MATERIALS TABLE




