# Supplementary material for: Prognostic value of microRNAs in hepatocellular carcinoma: a meta-analysis
Source: Oncotarget. 2017 Sep 14;8(63):107237–57. doi: 10.18632/oncotarget.20883 (PMC5739810; doi:10.18632/oncotarget.20883)
Supplement: Supplementary file 1 [file oncotarget-08-107237-s001.pdf]

# Prognostic value of microRNAs in hepatocellular carcinoma: a meta-analysis

## SUPPLEMENTARY MATERIALS

**Supplementary Table 1: Characteristics of studies estimating prognostic value of miRNA expression in hepatocellular carcinoma.** See Supplementary_Table_1
