# Supplementary material for: Prognostic value of microRNAs in hepatocellular carcinoma: a meta-analysis
Source: Oncotarget. 2017 Sep 14;8(63):107237–57. doi: 10.18632/oncotarget.20883 (PMC5739810; doi:10.18632/oncotarget.20883)
Supplement: Supplementary file 2 [file oncotarget-08-107237-s002.docx]

Supplementary Table 1: Characteristics of studies estimating prognostic value of miRNA expression in hepatocellular carcinoma.

| miRNA | Study | Country | Study  design | Sample | Number | Stage | Cut-off | Method | Follow-up  (month) | Result | HR  (L/H) | HR  (H/L) | 95%CI |
| --- | --- | --- | --- | --- | --- | --- | --- | --- | --- | --- | --- | --- | --- |
| 9 | Cai L, 2014 [5] | China | R | Tissue | 200 | I-IV | Median | RT-qPCR | 60 | OS^u^ |  | 2.01 | 1.41-2.86 |
| 9 | Sun J, 2015 [6] | China | R | Frozen | 60 | I-III | Median | qRT-PCR | 24 | DFS^m^ |  | 2.68 | 1.31-5.50 |
| 9 | Liu Y, 2017 [7] | China | R | Tissue | 120 | I-III | X-tile | qRT-PCR | 78 | OS^u^ |  | 3.41 | 1.60-7.24 |
|  |  |  |  |  |  |  |  |  |  | RFS^u^ |  | 2.37 | 1.29-4.35 |
| 21 | Tomimaru, 2010 [19] | Japan | R | Tissue | 30 | None | Median | qRT-PCR | 72 | OS^u^ |  | 1.13 | 0.35-3.61 |
| 21 | Karakatsanis, 2013 [20] | Greece | R | FFPE | 60 | I-IV | Mean | RT-qPCR | 90 | OS^u^ |  | 1.53 | 0.63-3.75 |
| 21 | He X, 2014 [21] | China | R | Frozen | 67 | I-IV | 1.50 | qRT-PCR | 44 | DFS^u^ |  | 3.03 | 0.67-13.60 |
| 21 | Wang WY, 2014 [22] | China | R | Frozen | 119 | I-IV | Median | qRT-PCR | 60 | OS^m^ |  | 3.19 | 1.91-10.01 |
|  |  |  |  |  |  |  |  |  |  | DFS^m^ |  | 5.90 | 3.01-13.76 |
| 21 | Chen WS, 2015 [2] | China | R | Tissue | 88 | I-III | None | RT-qPCR | 46.5 | DFS^m^ |  | 2.47 | 1.43-4.23 |
| 21 | Huang CS, 2015 [23] | China | R | Frozen | 112 | I-IV | Mean | qRT-PCR | 60 | OS^m^ |  | 2.28 | 1.39-7.92 |
| 21 | Shi KQ, 2015 [24] | TCGA | R | Frozen | 108 | I-IV | X-tile | Downloaded | 80 | OS^u^ |  | 1.42 | 1.06-1.90 |
| 21 | Hu L, 2016 [25] | China | R | Tissue | 32 | I-IV | None | qRT-PCR | 28 | OS^u^ |  | 3.22 | 1.15-9.02 |
| 22 | Zhang J, 2010 [26] | China | R | Frozen | 160 | I-III | Median | qRT-PCR | 48 | DFS^u^ | 1.52 |  | 0.92-2.51 |
| 22 | Zhou L, 2010 [27] | China | R | FFPE | 192 | I-IV | Median | qRT-PCR | 88 | OS^u^ | 2.34 |  | 1.55-3.54 |
| 22 | Chen M, 2016 [4] | TCGA | R | Tissue | 372 | I-IV | None | Downloaded | 60 | OS^m^ | 2.18 |  | 1.20-3.98 |
| 29a-5p | Zhu HT, 2012 [37] | China | R | Tissue | 218 | I-IV | Median | qRT-PCR | 81 | OS^u^ | 1.00 |  | 0.70-1.43 |
|  |  |  |  |  |  |  |  |  |  | RFS^m^ | 0.54 |  | 0.30-0.84 |
| 29a | Huang YH, 2012 [9] | China | R | Tissue | 216 | I-IV | None | RT-qPCR | 140 | OS^m^ | 2.49 |  | 0.90-6.85 |
|  |  |  |  |  |  |  |  |  |  | RFS^u^ | 1.19 |  | 0.82-1.74 |
| 29a | Parpart S, 2014 [38] | China | R | Tissue | 223 | I-III | Median | qRT-PCR | 66 | OS^u^ | 1.39 |  | 1.06-1.83 |
| 29c | Bae HJ, 2014 [40] | GEO | R | Tissue | 153 | I-IV | None | Downloaded | 60 | OS^u^ | 1.37 |  | 0.62-3.00 |
| 29c | Parpart S, 2014 [38] | China | R | Tissue | 223 | I-III | None | qRT-PCR | 66 | OS^u^ | 1.30 |  | 1.05-1.62 |
| 29c | Dong CW, 2016 [41] | China | R | Tissue | 91 | I-IV | Median | qRT-PCR | 60 | OS^m^ | 2.19 |  | 1.36-6.78 |
| 34a-5p | Li XY, 2015 [47] | China | R | Tissue | 114 | I-IV | None | ISH | 84 | OS^m^ | 1.81 |  | 1.03-3.18 |
|  |  |  |  |  |  |  |  |  |  | PFS^u^ | 1.22 |  | 0.92-1.62 |
| 34a | Yang F, 2013 [48] | China | R | Frozen | 30 | None | Median | qRT-PCR | 60 | OS^u^ | 3.54 |  | 1.67-7.52 |
| 34a | Cui X, 2015 [49] | China | R | Tissue | 120 | None | Median | qRT-PCR | 60 | OS^m^ | 1.44 |  | 1.13-1.72 |
|  |  |  |  |  |  |  |  |  |  | RFS^m^ | 1.49 |  | 1.15-1.79 |
| 34a | Xu X, 2015 [50] | China | R | Both | 75 | I-IV | Median | qRT-PCR | 60 | OS^m^ | 1.96 |  | 1.04-3.57 |
|  |  |  |  |  |  |  |  |  |  | DFS^m^ | 1.96 |  | 1.10-3.45 |
| 34c-3p | Xiao CZ, 2017 [52] | China | R | Tissue | 81 | I-III | None | qRT-PCR | 65 | OS^m^ |  | 1.95 | 1.06-3.57 |
|  |  |  |  |  |  |  |  |  |  | DFS^m^ |  | 1.84 | 1.00-3.38 |
| 34c | Xu X, 2015 [50] | China | R | Both | 75 | I-IV | Median | qRT-PCR | 60 | OS^m^ |  | 1.34 | 0.73-2.74 |
|  |  |  |  |  |  |  |  |  |  | DFS^m^ |  | 1.22 | 0.71-2.09 |
| 34c | Zhang X, 2016 [51] | China | R | Frozen | 80 | None | None | RT-qPCR | 72 | DFS^u^ |  | 0.84 | 0.71-0.97 |
| 148a | Heo MJ, 2014 [87] | Korea | R | Frozen | 59 | I-III | Median | qRT-PCR | 75 | OS^u^ | 6.59 |  | 1.77-24.58 |
|  |  |  |  |  |  |  |  |  |  | RFS^u^ | 1.70 |  | 1.01-2.86 |
| 148a | Li L, 2014 [88] | China | R | Tissue | X | I-IV | 1.00 | qRT-PCR | 107 | OS^u^ | 1.44 |  | 0.61-3.42 |
|  |  |  |  |  |  |  |  |  |  | RFS^u^ | 1.30 |  | 0.48-3.53 |
|  |  |  |  |  | 297-X |  | 2.00 |  |  | OS^u^ | 1.15 |  | 0.74-1.78 |
|  |  |  |  |  |  |  |  |  |  | RFS^u^ | 1.06 |  | 0.64-1.76 |
| 148a | Pan L, 2014 [89] | China | R | FFPE | 89 | I-IV | Median | RT-qPCR | ＞60 | RFS^u^ | 2.09 |  | 0.61-7.11 |
| 155-3p | Tang B, 2016 [98] | China | R | Frozen | 45 | I-IV | None | qRT-PCR | 60 | OS^u^ |  | 3.07 | 1.07-8.80 |
| 155 | Han ZB, 2012 [99] | China | R | FFPE | 100 | I-III | Median | qRT-PCR | 100 | OS^m^ |  | 4.74 | 2.33-9.62 |
|  |  |  |  |  |  |  |  |  |  | RFS^m^ |  | 2.75 | 1.28-4.91 |
| 155 | Huang YH, 2012 [9] | China | R | Tissue | 216 | None | None | RT-qPCR | 140 | RFS^m^ |  | 2.00 | 1.32-3.03 |
| 155 | Zhang L, 2016 [100] | China | R | FFPE | 124 | I-IV | Median | qRT-PCR | 60 | OS^u^ |  | 1.76 | 1.01-3.07 |
|  |  |  |  |  |  |  |  |  |  | DFS^u^ |  | 1.90 | 1.14-3.16 |
| 199a-5p | Morita K, 2015 [11] | Japan | R | Frozen | 70 | I-IV | Mean | qRT-PCR | 84 | RFS^u^ | 2.76 |  | 1.05-7.27 |
| 199a-5p | Li B, 2017 [113] | China | R | Frozen | 104 | I-III | Median | qRT-PCR | 78 | OS^m^ | 1.94 |  | 1.01-3.73 |
| 199a* | Wang P, 2015 [114] | China | R | FFPE | 135 | I-III | Mean | RT-qPCR | 50 | OS^u^ | 3.87 |  | 1.73-8.67 |
| 199a | Wang P, 2015 [114] | China | R | FFPE | 135 | I-III | Mean | RT-qPCR | 50 | OS^u^ | 3.12 |  | 1.73-5.63 |
| 200a | Xiao F, 2013 [116] | China | R | Tissue | 120 | I-III | Mean | qRT-PCR | 60 | OS^m^ | 2.62 |  | 1.12-4.64 |
| 200a | Feng J, 2015 [117] | China | R | Frozen | 115 | None | None | qRT-PCR | 108 | OS^m^ | 3.00 |  | 1.51-5.97 |
| 200a | Yang X, 2015 [118] | China | R | Tissue | 101 | 0-IV | None | qRT-PCR | 58 | OS^m^ | 2.48 |  | 1.49-4.13 |
| 203 | Chen HY, 2012 [119] | China | R | FFPE | 66 | I-III | Median | RT-qPCR | 100 | OS^m^ | 3.01 |  | 1.26-7.19 |
|  |  |  |  |  |  |  |  |  |  | RFS^m^ | 4.95 |  | 1.57-15.63 |
| 203 | Liu Y, 2015 [120] | China | R | FFPE | 95 | I-IV | Median | qRT-PCR | 68 | RFS^u^ | 0.91 |  | 0.29-2.88 |
| 203 | Wan D, 2016 [121] | China | R | Frozen | 138 | I-IV | Median | qRT-PCR | 60 | OS^m^ | 2.10 |  | 1.45-2.82 |
| 221 | Gramantieri, 2009 [132] | Italy | R | Frozen | 45 | None | Median | RT-qPCR | 120 | OS^u^ |  | 1.54 | 0.65-3.66 |
|  |  |  |  |  | 46 |  |  |  |  | RFS^u^ |  | 2.96 | 1.56-5.62 |
| 221 | Yoon SO, 2011 [133] | Korea | R | FFPE | 115 | I-III | Mean | RT-qPCR | 54.2 | RFS^u^ |  | 3.83 | 1.44-10.19 |
|  |  |  |  |  | 111 |  | 1.00 |  |  | MFS^m^ |  | 2.09 | 1.09-4.04 |
| 221 | Karakatsanis, 2013 [20] | Greece | R | FFPE | 60 | I-IV | Mean | RT-qPCR | 90 | OS^u^ |  | 1.31 | 1.03-1.67 |
| 221 | Rong M, 2013 [134] | China | R | FFPE | 48 | I-IV | Median | RT-qPCR | 23.3 | RFS^u^ |  | 1.36 | 0.91-2.02 |
| 221 | Chen F, 2016 [135] | China | R | FFPE | 135 | I-III | Median | qRT-PCR | 56 | OS^m^ |  | 2.97 | 1.63-5.41 |
| 21 | Tomimaru, 2013 [197] | Japan | R | Plasma | 126 | I-IIIA | 0.75 | qRT-PCR | ＞84 | RFS^u^ |  | 1.49 | 0.96-2.30 |
| 21 | Liu M, 2014 [198] | China | R | Serum | 136 | None | Median | qRT-PCR | 48 | OS^u^ |  | 1.43 | 1.08-1.89 |
| 21 | Wang X, 2015 [199] | China | R | Serum | 97 | I-IV | Median | qRT-PCR | 60 | OS^u^ |  | 2.38 | 1.34-4.23 |
| 21 | Cho HJ, 2017 [200] | Korea | R | Plasma | 120 | I-IV | Median | qRT-PCR | 130 | DFS^u^ |  | 0.83 | 0.55-1.25 |
| 29a-3p | Zhu HT, 2016 [202] | China | R | Serum | 74 | None | 1.37 | qRT-PCR | 70 | PFS^m^ |  | 2.17 | 1.11-4.23 |
| 29a | Cho HJ, 2017 [200] | Korea | R | Plasma | 120 | I-IV | Median | qRT-PCR | 130 | DFS^u^ |  | 0.60 | 0.37-0.99 |
| 122 | Köberle V, 2013 [195] | Germany | P | Serum | 295 | None | 25% | qRT-PCR | 26 | OS^u^ |  | 0.49 | 0.25-0.96 |
| 122 | Liu M, 2014 [198] | China | R | Serum | 136 | None | 6.88 | qRT-PCR | 48 | OS^u^ |  | 1.24 | 1.06-1.46 |
| 122 | Cho HJ, 2015 [205] | South Korea | R | Plasma | 120 | I-IV | 75% | qRT-PCR | 96 | OS^u^ |  | 1.89 | 0.70-5.00 |
|  |  |  |  |  |  |  |  |  |  | DFS^u^ |  | 1.06 | 0.82-1.37 |
| 122 | Xu Y, 2015 [206] | China | R | Serum | 122 | None | Median | qRT-PCR | ＞40 | OS^u^ |  | 0.26 | 0.14-0.47 |
| 122 | Kim SS, 2016 [207] | South Korea | R | Plasma | 161 | I-IV | 100.00 | qRT-PCR | 79 | OS^m^ |  | 1.19 | 0.76-1.85 |
| 122 | Ng KT, 2016 [208] | China | R | Plasma | 62 | None | ROC | RT-qPCR | ＞125 | OS^m^ |  | 7.24 | 0.56-93.42 |
|  |  |  |  |  |  |  |  |  |  | DFS^m^ |  | 5.47 | 0.58-51.77 |
| 148a | Ng KT, 2016 [208] | China | R | Plasma | 62 | None | ROC | RT-qPCR | ＞125 | OS^m^ | 2.22 |  | 0.16-33.33 |
| 148a | Wang F ,2016 [211] | China | R | Serum | 76 | I-IV | Median | qRT-PCR | 36 | OS^m^ | 2.26 |  | 1.08-4.72 |
| 192-5p | Zhu HT, 2016 [202] | China | R | Serum | 74 | None | 2.24 | qRT-PCR | 72 | OS^u^ |  | 2.71 | 1.50-4.91 |
|  |  |  |  |  |  |  |  |  |  | PFS^m^ |  | 2.17 | 1.11-4.23 |
| 192 | Ng KT, 2016 [208] | China | R | Plasma | 62 | None | ROC | qRT-PCR | ＞125 | OS^m^ |  | 0.67 | 0.05-8.68 |
|  |  |  |  |  |  |  |  |  |  | DFS^m^ |  | 0.50 | 0.03-7.50 |
| 224-5p | Liu M, 2014 [198] | China | R | Serum | 136 | None | Median | qRT-PCR | 48 | OS^u^ |  | 1.43 | 1.13-1.81 |
| 224 | Zhuang LP, 2014 [218] | China | R | Serum | 182 | None | Median | qRT-PCR | 90 | OS^m^ |  | 2.09 | 1.14-3.81 |

HR (L/H): hazard ratios of low expression versus high expression of miRNAs; HR (H/L): hazard ratios of high expression versus low expression of miRNAs; CI: confidence intervals; TCGA: Tumor Cancer Genome Atlas; GEO: Gene Expression Omnibus; R: retrospective; P: prospective; FFPE: formalin-fixed paraffin-embedded; ROC: receiver operating characteristic; qRT-PCR: quantitative real-time polymerase chain reaction; RT-qPCR: reverse transcription quantitative real-time polymerase chain reaction; ISH: in situ hybridization; OS: overall survival; DFS: disease-free survival; RFS: recurrence-free survival; PFS: progression-free survival; MFS: metastasis-free survival; ^u^Univariate analysis; ^m^Multivariate analysis. In order to facilitate read and statistics, studies estimating prognostic value of different miRNAs are shown in blue and white; studies which cannot be merged are shown in yellow.
